# ^18^ F-Fluorodeoxyglucose Uptake in Bilateral Diaphragmatic Crura: A Relatively Uncommon Benign Variant Noted in a Treated Case of Extraosseous Paraspinal Ewing's Sarcoma

**DOI:** 10.1055/s-0044-1779284

**Published:** 2024-02-06

**Authors:** Parth Baberwal, Sunita N. Sonavane, Sandip Basu

**Affiliations:** 1 Radiation Medicine Centre, Bhabha Atomic Research Centre, Tata Memorial Hospital Annexe, Parel, Mumbai, Maharashtra, India; 2 Homi Bhabha National Institute, Mumbai, Maharashtra, India

**Keywords:** extraosseous paraspinal Ewing's sarcoma, bilateral diaphragmatic crura, FDG-PET/CT, benign variant, nonspecific FDG uptake

## Abstract

A toddler was diagnosed with extraosseous Ewing's sarcoma, primary large epidural paraspinal soft tissue in the lumbar region encasing the cord and neural foramen from D12–L1 to L4–L5. After eight cycles of induction chemotherapy with vincristine, doxorubicin, and cyclophosphamide alternating with etoposide and ifosfamide, ^18^ F-FDG positron emission tomography/computed tomography ( ^18^ F-FDG-PET/CT) scan confirmed no active disease. Later external beam radiotherapy (EBRT) at D10–L5 was completed. At 3 months follow-up, ^18^ F-FDG-PET/CT reconfirmed no residual/active disease; however, a new incidental finding of diffuse benign bilateral diaphragmatic ^18^ F-FDG uptake was noted in the clinically asymptomatic patient, which remained unexplained.

## Case Report

This case demonstrates a relatively uncommon benign variant of bilateral ^18^ F-FDG ( ^18^ F-fluorodeoxyglucose) uptake of in the diaphragmatic crus in a case of Ewing's sarcoma.

**Fig. 1:**
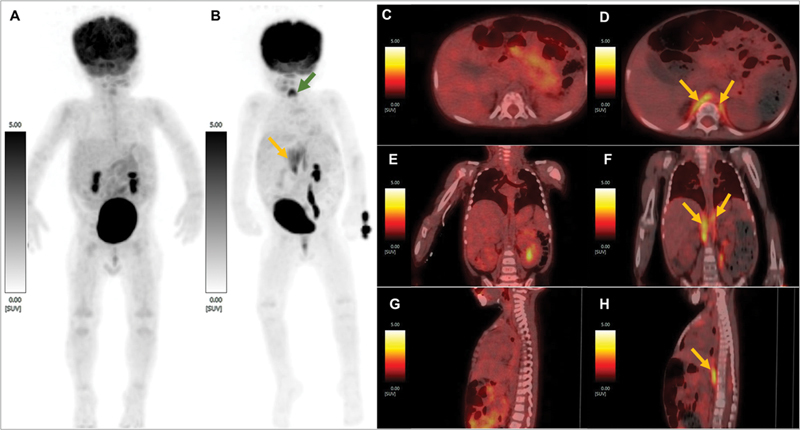
( **A** ) Maximum intensity projection (MIP) of ^18^ F-FDG positron emission tomography ( ^18^ F-FDG-PET) post-induction chemotherapy showing no abnormal tracer uptake in the whole-body survey and ( **B** ) MIP of ^18^ F-FDG PET done 3 months after radical radiotherapy to the D10 -L5 vertebrae and para-spinal tissue showing bilateral diaphragmatic crural uptake of tracer (maximum standardized uptake value [SUVmax] = 3.39; marked with yellow arrows) done for response assessment post treatment (post-induction, consolidation chemotherapy and radiotherapy). Column of images showing ( **C** ) axial, ( **E** ) coronal, and ( **G** ) sagittal views of fused post induction chemotherapy ^18^ F-FDG-PET/CT and ( **D** ) axial, ( **F** ) coronal, and ( **H** ) sagittal views of post treatment ^18^ F-FDG-PET/CT images, respectively showing similar findings as mentioned above. No diaphragmatic elevation was noted and the patient did not complain of any respiration-related issues. In MIP ( **B** ) a note is made of new-onset tracer uptake at the site of vocal cords (marked with *green arrow* ).

A 2-year-old boy initially presented with reduced frequency of passage of stools and urine with sudden onset loss of bilateral lower limb movements. Magnetic resonance imaging (MRI) was done, which showed large epidural soft tissue encasing the cord and neural foramen from D12–L1 to L4–L5. Computed tomography (CT) guided biopsy of the soft-tissue lesion showed Ewing's sarcoma of the lumbar paraspinal region. He had undergone 8 cycles of induction chemotherapy with vincristine, doxorubicin, and cyclophosphamide alternating with etoposide and ifosfamide. The patient then underwent ^18^ F-FDG positron emission tomography/CT (PET/CT) scan, which showed no active disease ( Fig. 1 ). The patient then underwent an external beam radiotherapy (EBRT) of 45 Gy in 25 fractions to D10–L5. Further in asymptomatic clinical status, at 3 months of follow-up, ^18^ F-FDG PET/CT reconfirmed no residual/active disease. Additionally, a new incidental finding of diffuse increased tracer uptake in the bilateral diaphragmatic crus (maximum standardized uptake value of 3.39) was noted with no definite CT changes.

## Discussion

Ewing's sarcoma is the second most common malignancy of bone in children and adolescents. Extraosseous Ewing's sarcoma (EES) is rare in comparison with Ewing's sarcoma of the bone (constitutes ∼15%), reported with equal frequency in both males and females. The most common location for EES is the paravertebral soft-tissue mass, which can extend to the spinal epidural space, as noted in this case. 1 2

^18^ F-FDG has been used for disease staging to guide management by monitoring the patient during or at the end of therapy and to restage a patient suspected of recurrence. 3 4 Albeit it offers high sensitivity, nonspecific uptake may hinder accurate interpretation and requires that the PET reader be able to differentiate such spurious findings and not be nonchalant in reporting it as it may misguide management. Careful history-taking and clinical examination are important as correlation of it with such findings can help in more accurate interpretation. Many spurious findings have been noted, especially in the settings of post-therapeutic interventions like surgery, chemotherapy, and radiotherapy (RT). 5 Diaphragmatic crural uptake (unilateral or bilateral) is a rare finding noted due to a multitude of reasons. One of the case reports showed that the patient developed right hemidiaphragm paralysis after demonstrating right-sided unilateral crus of the diaphragm ^18^ F-FDG uptake, sometime after RT to neuroendocrine carcinoma of the right lung. 6 Such similar incidences of diaphragmatic paralysis have been noted after RT. 7 8 9

A known cause of ^18^ F-FDG uptake in muscle has been voluntary muscle activation such as exercise, labored breathing, muscle spasms, etc. Joshi and Lele have reported a case of right lung carcinoma showing increased ^18^ F-FDG uptake in the left crus of the diaphragm secondary to the right hemidiaphragm paralysis, possibly due to compensatory overactivity of the left diaphragm. 10 Similarly, there have been a reported incidence of unilateral crural uptake on ^18^ F-FDG PET due to increased breathing, which was hypothesized due to increased work of breathing of the right lung secondary to restricted motion of the left hemidiaphragm due to metastatic pleural disease of the left side. 11

Albeit our patient underwent RT for Ewing's sarcoma of the paraspinal region, our patient was clinically asymptomatic at the time of ^18^ F-FDG scan and never had any breathing difficulties till 3 months post scan as opposed to scans elaborated by Jolepalem et al, 6 wherein benign unilateral uptake in the diaphragmatic crus was described. In case of unilateral diaphragmatic crus uptake, when the uptake is contralateral to the paralyzed hemidiaphragm, the explanation is a compensatory increase in the work of the functioning side, while for uptake on the ipsilateral side, phrenic nerve neuropathy is implicated. 6

In the presented case, there were no such findings that would point toward hemidiaphragmatic paralysis. The only incidental finding in our case was increased tracer uptake in the vocal cords. Bilateral diaphragmatic crural FDG uptake was noted in the cases reported by Joseph et al 12 and Park et al. 13 In the above-mentioned cases, the child was agitated and crying at the time of uptake. This was associated with increased uptake in the vocal cord, the entire diaphragm and diaphragmatic crura, accessory muscles associated with respiration and tongue muscles secondary to vocalization, and hyperventilation and hyperactivity of muscles at all these locations. However, from the patient's history, our patient was sedated throughout the uptake and scan acquisition phases and no uptake was noted in the rest of the diaphragm and the accessory muscles, as opposed to the case presented by Joseph et al 12 and Park et al. 13

## Conclusion

In this clinically asymptomatic case of paraspinal EES with ^18^ F-FDG-PET/CT scan showing findings of bilateral crural uptake following RT-CT, the patient didn't have any breathing difficulties till 3 months post-scan, thus, the cause of uptake in diaphragmatic crura was apparently unexplained in the absence of uptake in other voluntary muscles in the torso.
